# MicroRNAs and cell fate in cortical and retinal development

**DOI:** 10.3389/fncel.2013.00141

**Published:** 2013-09-03

**Authors:** Federico Cremisi

**Affiliations:** Scuola Normale SuperiorePisa, Italy

**Keywords:** cortex, retina, cell-fate, heterochronic, timing, cell birth date, development

## Abstract

MicroRNAs (miRNAs) are involved in crucial steps of neurogenesis, neural differentiation, and neuronal plasticity. Here we review experimental evidence suggesting that miRNAs may regulate the histogenesis of the cerebral cortex and neural retina. Both cortical and retinal early progenitor cells are multipotent, that is, they can generate different types of cortical or retinal cells, respectively, in one lineage. In both cortical and retinal development, the precise timing of activation of cell fate transcription factors results in a stereotyped schedule of generation of the different types of neurons. Emerging evidence indicates that miRNAs may play an important role in regulating such temporal programing of neuronal differentiation. Neuronal subtypes of the cortex and retina exhibit distinct miRNA signatures, implying that miRNA codes may be used to specify different types of neurons. Interfering with global miRNA activity changes the ratio of the different types of neurons produced. In fact, there are examples of cell fate genes that are regulated at the translational level, both in retinogenesis and in corticogenesis. A model depicting how miRNAs might orchestrate both the type and the birth of different neurons is presented and discussed.

**Glossary**.

• Lineage: the temporally ordered cell progeny of an individual progenitor cell.

• Specification: the (reversible) process by which a cell becomes capable of, and biased toward, a particular fate.

• Commitment: the process by which cell fate is fully determined and can no longer be affected by external cues.

• Potency: the entire complement of cells that a progenitor can ultimately produce.

• Multipotency: the ability to give rise to more than one cell type.

• Progenitor: a dividing cell that, in contrast to a stem cell, cannot proliferate indefinitely.

• Antago-miR: modified antisense oligonucleotide that blocks the activity of a miRNA.

• Heterochronic neuron: type of neurons that is generated at inappropriate times of development.

• Neuron birth date: the time of the last mitosis of a neuronal cell.

## GENERAL IMPLICATIONS OF miRNAs IN NEURAL DEVELOPMENT

MicroRNAs (miRNAs) are a large family of non-coding RNAs of approximately 21 nucleotides in length, which inhibit gene expression at the translational level and are involved in the control of many developmental and cellular processes in eukaryotic organisms, including vertebrate neural development (Krol et al., 2010). miRNAs have been found to regulate many aspects of neural development, including the early steps in neurogenesis, the specification and differentiation of neural progenitor cells, brain patterning, and the plasticity of mature neurons (Coolen and Bally-Cuif, 2009; Fineberg et al., 2009; Bian and Sun, 2011).

Examples of miRNAs involved in the specification of distinct types of mature neurons have also been described. miR-7a is expressed in a gradient opposing Pax6 along the ventricular walls and restricts its translation in the dorsal aspect. *In vivo* inhibition of miR-7a in Pax6-negative regions of the lateral wall induced Pax6 protein expression and increased dopaminergic neurons in the olfactory bulb (De Chevigny et al., 2012). miR-132 plays a key role in the differentiation of dopamine neurons by directly regulating the expression of Nurr1, which is one of the most important transcription factors in determining dopamine neuron development and differentiation (Yang et al., 2012). The overexpression of miR-181a and miR-125b increases the expression of dopaminergic markers and the ratio of tyrosine hydroxylase (TH) positive neurons generated by neural stem cells derived from human embryonic stem cells, whereas the inhibition of these miRNAs impairs the generation of the dopaminergic subtype (Stappert et al., 2013). miR-9, which is reiteratively used in patterning, neurogenesis, and differentiation (Coolen and Bally-Cuif, 2009), also has a role in establishing distinct types of motor neurons. miR-9 is transiently expressed in a motor neuron subtype together with its target gene FoxP1, which determines distinct motor neuron subtypes. Consequently, miR-9 overexpression or knockdown switches columnar identities in developing chick spinal cords (Otaegi et al., 2011).

Recent observations suggest that combinatorial miRNA expression may contribute to specifying neuron identity. The expression of a large fraction of known miRNAs with distinct expression profiles in glutamatergic and subtypes of GABAergic neurons has recently been demonstrated (He et al., 2012). In the mouse retina, a comprehensive survey of miRNA expression was achieved by *in situ* hybridization, revealing the expression of specific sets of miRNAs in distinct neuronal subtypes (Karali et al., 2010). Here we discuss the role that miRNAs may play in the generation of distinct types of neurons at different times in the development of layered structures. We will focus on the histogenesis of the neural retina and the cerebral cortex, where the role of miRNAs has been most widely investigated.

## CORTICOGENESIS AND RETINOGENESIS SHOW SIMILAR MECHANISMS FOR ESTABLISHING DISTINCT CELL FATES

**FIGURE 1:**
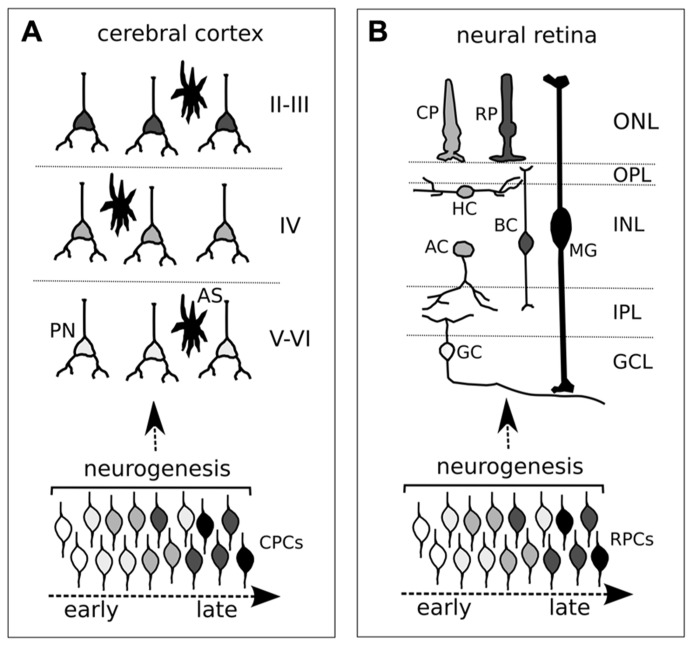
**Neurogenic timing in the developing cortex (A) and retina (B). (A,B)** Different degrees of gray depict distinct neuronal identities in cortex **(A)** and retina **(B)**. Both cortical and retinal progenitor cells (CPCs and RPCs, respectively) change competence over time (different degrees of gray from early to late). Although an overlap in neuronal cell birth periods is shown, the time of exit from the cell cycle (neurogenesis, or cell birth date) influences the acquisition of distinct cell identities of post-mitotic neurons. **(A)** CPCs comprise both ventricular (primary) and subventricular (secondary) progenitor cells. PN, projecting neuron; AS, astrocyte. Roman numerals indicate cortical layers. **(B)** Different retinal neurons and glia. CP, cone photoreceptor; RP, rod photoreceptor; HC, horizontal cell; BC, bipolar cell; MG, Müller glia; AC, amacrine cell; GC, ganglion cell; ONL, outer nuclear layer; OPL, outer plexiform layer; INL, inner nuclear layer; IPL, inner plexiform layer; GCL, ganglion cell layer.

One main characteristic of the both retina and the cortex is that the identity of a certain type of mature neuron correlates with the time of its last division (cell birth date). Cortical projection neurons are derived from progenitor cells of the dorsal forebrain. After an initial phase of expansion, which is realized by symmetric divisions, progenitor cells of the ventricular zone (radial glia) start asymmetric divisions that generate new radial glia and either post-mitotic neurons (direct neurogenesis) or secondary (intermediate) progenitors. The net result is that the pool of progenitors does not deplete over the time and a single progenitor can generate a lineage made of different types of neurons with different birth dates. In the cortex, neurons with early birth dates are produced by primary (early) progenitor cells of the ventricular zone (radial glia) and populate the deep layers VI–V. Neurons with late birth dates, which fill the superficial layers II–III, are primarily generated by Tbr2-positive secondary progenitor cells of the subventricular zone (Leone et al., 2008; Sessa et al., 2008, 2010; **Figure 1A**). By the time a young neuron has progressed through its final mitotic division, the cell has acquired the information needed to migrate to the layer typical of its birth date, independent of the environment. Cellular studies by transplantation experiments suggest a progressive restriction in the developmental potential of cortical cells. Early progenitors, which normally produce deep-layer neurons, are multipotent: these cells can directly produce upper-layer neurons when transplanted into an older brain environment (McConnell and Kaznowski, 1991). Conversely, the progenitors of layer IV–II neurons have lost the ability to form layer VI neurons if transplanted into younger brains (Frantz and McConnell, 1996; Desai and McConnell, 2000).

In the retina, landmark studies of lineage-tracing have shown that early progenitor cells are multipotent and, likewise, early cortical progenitors can generate lineages containing different types of neurons (Turner and Cepko, 1987; Holt et al., 1988; Wetts and Fraser, 1988). The six types of neurons and the Müller glia making up the vertebrate retina are generated in a stereotyped sequence, with a correlation between cell birth date and cell fate, though with some overlap in the production of retinal cell types at any given time. Retinal ganglion cells (RGCs) are generated first, followed by the production of cone photoreceptors, horizontal cells, and amacrine neurons. Rod photoreceptors, bipolar neurons, and Müller glia are generated last (**Figure 1B**). Retinal progenitors generate these different cell types by proceeding through intrinsically defined competence states, with a certain degree of influence of environmental cues.

A growing list of transcription factors has emerged as key intrinsic regulators of cortical and retinal cell fate. Cortical progenitors sequentially activate a number of transcription factor genes that have the potential to determine the fates of their daughter cells. Early progenitor cells produce deep-layer neurons that express Fezf2 and Ctip2, which specify subcortically projecting neurons. Late progenitors generate upper-layer neurons expressing Satb2, which is required for the formation of axonal projections that connect the two cerebral hemispheres. Fezf2/Ctip2 and Satb2 pathways appear to be mutually repressive, thus ensuring that individual neurons adopt either a subcortical or callosal projection neuron identity (Leone et al., 2008). The molecular nature of this cross-repression is under scrutiny (Srinivasan et al., 2012). Interestingly, the Satb2 protein, in contrast to mRNA, was not detected in late progenitors, but was detected in post-mitotic cells of the cortical plate, suggesting that a Satb2 translation block might occur in the progenitor cell (Britanova et al., 2005).

Retinal cell fate specification is mainly regulated by combinations of bHLH and homeobox genes. In mice, Atoh7 (bHL) and Pou4f2 (homeobox) cooperate to regulate RGC genesis. The expression of Prox1 (homeobox) is essential for horizontal cell generation, while a number of factors, including Neurod1 and Neurod4 (bHL), Pax6 and Six3 (homeobox), regulate the production of amacrine cells. Crx (homeobox) is crucial for specifying photoreceptors, and Vsx2 (also named Chx10, homeobox) is required for bipolar cell genesis (Ohsawa and Kageyama, 2008). Notably, the *Xenopus* homologs of Crx and Vsx2 (Xotx5b and Xvsx, respectively) coordinate the production of photoreceptors and bipolar cells via a translational control mechanism (Decembrini et al., 2006). The sequential expression of the two Sry-related HMG box proteins Sox11 and Sox4, during retinogenesis, leads to the fine adjustment of retinal differentiation. Overexpression of Sox11 and Sox4 in retinal progenitors increases the number of cone cells and dramatically decreases the number of rod cells and Müller glia, by acting through epigenetic mechanisms (Usui et al., 2013).

Although key transcription factors of cell fate are known, how they are activated in distinct cells at specific developmental times is not clear. Consequently, the mechanisms responsible for shifts in competence over time in the lineage of a progenitor cell remain largely elusive. One important feature shared by the cortex and retina is that the potency of progenitor cells diminishes and their competence changes as they “age” during embryonic development. We do not know the precise sort of “clock” that measures a progenitor’s age, though one possible way would be through the length of its cell cycle. In fact, during neural development the proliferation rate decreases over time as the progenitor cell cycle length increases (Caviness et al., 1995; Alexiades and Cepko, 1996; Decembrini et al., 2006).

The proliferation rate of neural progenitor cells is regulated by the activation of a number of growth factor pathways. The activation of Wnt and fibroblast growth factor (FGF) pathways during cortical development supports the expression of cyclinD1 and shortens the cell cycle of progenitors, thus promoting proliferation, expansion of apical progenitors, and reduced generation of basal progenitors (Salomoni and Calegari, 2010). Wnts and FGFs, together with bone morphogenic proteins (BMPs), play a crucial role also in cortical patterning (Rubenstein, 2011) but they have not been shown to directly affect the establishment of distinct neuronal fates. The Shh pathway supports cell cycle progression, both in the retina (Wang et al., 2005; Locker et al., 2006) and in the mouse cerebral cortex (Komada et al., 2008). Interestingly, blocking the Shh pathway affects the histogenesis of both the *Xenopus* retina (Decembrini et al., 2009) and mouse cortex (Komada et al., 2008). In the *Xenopus* retina, this is caused by release from translational inhibition of Xotx5b and Xvsx, which are necessary for specifying the bipolar fate. Notably, shortening the cell cycle by E2F overexpression exerts opposite effects, thus supporting the idea that Shh acts on cell fate through the cell cycle machinery (Decembrini et al., 2006). Whether (and how) cell cycle progression relates to the clock controlling the competence of differentiation, and how this clock in turn regulates activation of the transcription factors that specify the distinct neuron types remain open issues.

## miRNAs AND CORTICAL HISTOGENESIS

Most of our knowledge on the role of miRNAs in cortical and retinal histogenesis comes from analyzing the phenotypes observed after global loss of miRNA regulation, which is induced by disrupting the pre-miRNA processing enzyme Dicer. Conditional knock-out (CKO) of Dicer in the cortex was achieved after breeding Dicer:lox/lox mice with distinct forebrain Cre-driver mouse strains, including Nestin:Cre, Emx1:Cre, or FoxG1:Cre (De Pietri Tonelli et al., 2008; Kawase-Koga et al., 2009; Nowakowski et al., 2011; **Table 1**). A general effect common to different mouse strains driving early inactivation of Dicer in the cortex is the induction of cell death, because miRNAs target several players of the DNA-damage response signal-transduction network (Bailey et al., 2010). However, Dicer CKO also has profound effects on cortical layering.

**Table 1:** Dicer-CKO phenotypes in the cortex and retina.

Cre transgene	Cre expression	Main phenotype	Reference
**Cortex**
Foxg1-Cre	From E8 in most forebrain cells	Altered balance of apical and basal progenitors	Nowakowski et al. (2011)
Emx1-Cre	From E10 to E10.5 in most cells of the dorsal telencephalon	Overproduction of early-born neurons and reduced number of Brn1-expressing upper-layer neurons	Kawase-Koga et al. (2009)
Nestin-Cre	From E10 to E10.5 in forebrain stem cells and progenitors	Affected late-born neuron generation and migration	De Pietri Tonelli et al. (2008)
CamKII-Cre	From E15.5 in post-mitotic neurons of the cortex and hippocampus	Normal layering; reduced dendritic branch elaboration	Davis et al. (2008)
**Retina**
Chx10-Cre	Mosaic pattern, from E14.5 in progenitors of all retinal layers	Decreased ERG responses, retinal disorganization, progressive retinal degeneration from P16	Damiani et al. (2008)
αPax6-Cre	Peripheral retina from day E10.5; differentiated amacrine cells, by E14.5	Overproduction of ganglion cells, failure to generate late cell types such as Müller glia and rod photoreceptors	Georgi and Reh (2010)
Dkk3-Cre	From E10.5 in progenitors of all neuroretinal cell types	Microphthalmia, massive apoptosis	Iida et al. (2011)
Rx-Cre	Ubiquitously in the developing neuroretina and optic stalk; later in the optic chiasm	Microphthalmia, massive apoptosis, defects in retinal ganglion cell axon pathfinding	Pinter and Hindges (2010)

FoxG1:Cre;Dicer:lox/lox embryos deactivate Dicer from E8, and the effects on the expression of mature miRNAs are detectable by E11.5 in most forebrain cells. In these mice, neuroepithelial stem cell identity is not affected, but expression of the markers of radial glia Nestin, Sox9, and ErbB2 is abnormally low. Early telencephalic progenitors generate correct proportions of neurons after Dicer deletion, but many of those neurons migrate abnormally, possibly due to a defect in radial glia-guided migration. Moreover, the population of secondary (basal) progenitors, which are generated by the radial glia, is disorganized and expanded (Nowakowski et al., 2011). The depletion of miR-92b may play a crucial role in generating this phenotype. In fact, this miRNA is predicted to target the 3′ untranslated region (UTR) of the transcription factor Tbr2, which regulates the generation of intermediate progenitors. Acute miR-92b gain of function causes rapid reductions in the ratio of Tbr2-expressing cells, whereas acute miR-92b loss of function has opposite effects (Nowakowski et al., 2013).

Dicer CKO in dorsal forebrain cells has been achieved with Cre expression from around E10 to E10.5 in Emx1:Cre;Dicer:lox/lox and Nestin:Cre;Dicer:lox/lox mice. The Nestin:Cre strain drove a milder and later inactivation of Dicer as compared to the Emx1:Cre strain. Emx1:Cre;Dicer:lox/lox showed overproduction of early-born neurons and a reduced number of Brn1-expressing upper-layer neurons as compared with controls, and the remaining ones were intermingled with Tbr1-expressing deep-layer neurons. Nestin:Cre;Dicer:lox/lox mice had no defects in the production of early-born neurons, but exhibited affected generation and migration of late-born neuron (De Pietri Tonelli et al., 2008; Kawase-Koga et al., 2009).

Dicer CKO in post-mitotic neurons of CamKII:Cre;Dicer:loxP/loxP mice caused reduced dendritic branch elaboration, but generated normal cortical layering (Davis et al., 2008), indicating that a late inactivation of Dicer cannot affect layer identity.

Altogether, these results show that mature miRNAs are required at different times in corticogenesis to fine-tune cell fate and, depending on the time of Dicer inactivation, different cell types and layers are affected. Unfortunately, these studies did not address the question of whether the translation of key transcription factors of cortical cell fate was affected.

## miRNAs AND RETINAL HISTOGENESIS

Four different Dicer-CKO mouse models have recently allowed investigating the effects of global miRNA down-regulation in mouse retinal development (**Table 1**). Cre-mediated Dicer excision in retinal progenitors resulted in phenotypes of variable severity, likely dependent on the time and the extent of Dicer deletion. Accordingly, when Dicer excision began earlier in retinal development, or when Cre was more uniformly expressed throughout the developing retina, more severe phenotypes were consistently observed. Likewise, when driving Dicer CKO in the developing cortex, a general effect of cell death was observed at different extents and times in all the retina CKOs (Damiani et al., 2008; Pinter and Hindges, 2010; Iida et al., 2011; Nowakowski et al., 2013).

Chx10-Cre expression exhibits a mosaic pattern and begins before embryonic day 14.5 in progenitors of all retinal layers. Dicer CKO driven by the Chx10-Cre transgene led to decreased electroretinogram (ERG) responses, morphological anomalies, and formation of photoreceptor rosettes at post-natal day 16. This phenotype progressed to more general cellular disorganization and widespread degeneration of retinal cell types as the animals aged (Damiani et al., 2008).

αPax6-Cre is expressed in peripheral regions of the developing retina, beginning on embryonic day 10.5. Dicer CKO driven by αPax6-Cre, which inactivated Dicer in a less mature population of retinal progenitors than Chx10-Cre, generated a more severe phenotype, consisting in the abnormal differentiation of retinal cell types. The production of early generated cell types (RGC and horizontal cells) was increased. Interestingly, ganglion cells (GCs) were generated beyond their normal competence window and, probably as a consequence, the Dicer-deleted areas of the retina showed a decrease in later generated cell types (amacrine cells and rod photoreceptors). These results indicate that miRNAs are required for shifts in the competence of retinal progenitors over time (Georgi and Reh, 2010).

Dkk3-Cre is ubiquitously expressed in all retinal progenitors beginning on embryonic day 10.5. Dicer CKO by this transgene produced massive death of retinal progenitor cells (RPCs), resulting in microphthalmia and the absence of layers. *In vitro* reaggregation culture of Dicer-CKO retinal cells revealed that cell death and the suppression of proliferation by Dicer inactivation occurred in a cell-autonomous manner (Iida et al., 2011). Such results are consistent with the phenotype observed after early inactivation of Dicer by morpholino microinjection in *Xenopus* (Decembrini et al., 2008).

Rx-Cre is ubiquitously expressed in the developing neuroretina. Dicer CKO by Rx-driven Cre activation caused cell death and a reduction in overall eye size. However, a RGC layer formed and no defects were observed in the formation of the optic disc, which is the exit point for RGC axons from the retina. Interestingly, mutants showed a marked increase in ipsilateral projections, with RGC axons extending outside the optic chiasm or showing aberrant projections, indicating a miRNA role in ensuring correct axon guidance decisions. Notably, these phenotypes were not the result of a mis-patterning of the eye (or the chiasm), suggesting that miRNAs have direct functions in the intracellular processes needed for axon growth and pathfinding (Pinter and Hindges, 2010).

Recent observations suggest that distinct miRNAs might be responsible for the cell death observed after Dicer CKO. In *Xenopus*, the inhibition of miR-24a, which is predicted to target the pro-apoptotic factors caspase-9 and protease-activating factor 1 (apaf1), resulted in increased apoptosis of retinal progenitors and microphthalmia (Walker and Harland, 2009). In mice, the knock-out of miR-124 caused apoptosis of newly differentiated cone photoreceptors (Sanuki et al., 2011). Individual miRNAs controlling retinal cell identity are emerging. miR-204 has an active role in establishing dorsoventral (D/V) polarity of the optic cup of medaka fish. When miR-204 activity was blocked by antago-miR, the expression domain of ventral markers was reduced or absent, whereas the expression domains of the dorsal markers were expanded ventrally. A reciprocal molecular phenotype was observed after miR-204 overexpression. These phenotypes were associated with concomitant up- or down-regulation of olMeis2, which is a target of miR-204 and mediates its effects on D/V eye polarity (Conte et al., 2010).

## DISTINCT miRNAs AND mRNAs REGULATE THE TIMING OF RETINOGENESIS

**FIGURE 2:**
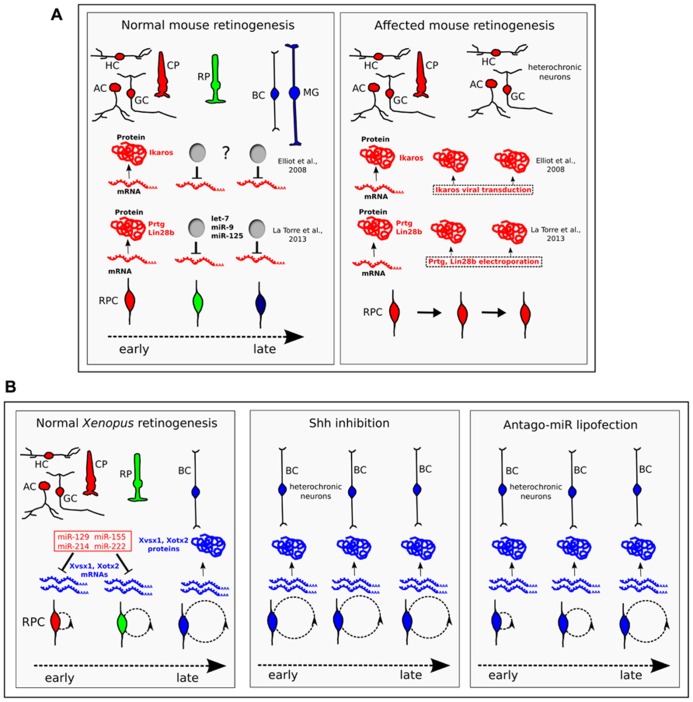
**The temporal identity of retinal progenitor cells (RPCs) is defined through the translational regulation of key proteins.** **(A)** In mice, Ikaros, Prtg, and Lin-28b are transcribed throughout retinogenesis, but are translated only in early RPCs. While the molecular nature of the inhibitor of Ikaros translation (“?” label) is unknown, Prtg and Lin-28b are targeted by let-7, miR-9, and miR-125. When the protein expression of Ikaros, or Prtg and Lin-28b, is forced throughout retinogenesis, heterochronic neurons of the early-born type (HC, horizontal cells; AC, amacrine cells; GC, ganglion cells) are generated at late times in retinogenesis (Elliott et al., 2008; La Torre et al., 2013). CP, cone photoreceptor; RP, rod photoreceptor; BC, bipolar cell; MG, Müller glia. **(B)** In *Xenopus*, bipolar fate is driven by the homeobox Xvsx1 and Xotx2 genes, which are transcribed in RPCs from early developmental stages (15 and 25, respectively), but are translated only from late stages 37 and 38–39, respectively (Decembrini et al., 2006). A set of four cell cycle-regulated miRNAs (miR-129, miR-155, miR-214, and miR-222, in red) bind the 3′UTR of Xvsx1 and Xotx2, inhibiting their translation in early RPCs. In normal *Xenopus* retinogenesis, the duration of the cell cycle (indicated by dashed circles) inversely correlates with the expression of the four miRNAs. Lengthening the cell cycle by treatment with the Shh signaling inhibitor cyclopamine (Shh inhibition) down-regulates this set of miRNAs, leads to earlier translation of Xvsx1 and Xotx2 and causes the generation of heterochronic bipolar cells. Antago-miR lipofection in early RPCs inhibits the activity of the four miRNAs. Compared to cyclopamine treatment, the lipofection exerts similar effects on the translation of Xvsx1 and Xotx2, and on the generation of bipolar cells, but does not affect progression of the cell cycle (Decembrini et al., 2009). This favors the hypothesis that cell cycle progression may affect neuronal fate through the set of four miRNAs. In these experiments, the effect of miRNAs on Müller glia was not examined. CP, cone photoreceptor; RP, rod photoreceptor; HC, horizontal cell; BC, bipolar cell; AC, amacrine cell; GC, ganglion cell.

A defined temporal sequence of gene expression that could explain the chronological order of cell birth in different neuronal lineages was first described in *Drosophila* (Isshiki et al., 2001). Further studies have confirmed the generality of this strategy, with different sequences of transcription factors being used in different structures of the *Drosophila* nervous system to generate neuronal diversity, according to a well-defined time schedule (Bayraktar and Doe, 2013; Li et al., 2013; Suzuki et al., 2013). Homologs of key transcription factors defining the temporal identity of *Drosophila* neuroblasts have now been detected in the developing mammalian retina. One of them, IKAROS family zinc finger 1 (Ikzf1/Ikaros), is a mouse ortholog of hunchback (hb), which is necessary and sufficient to specify early-born neurons in *Drosophila*. Ikaros is both necessary and sufficient to confer early temporal competence to mouse RPCs. In fact, mis-expression of Ikaros is sufficient to generate early-born neurons at inappropriate times: after viral Ikaros transduction in late RPCs, heterochronic amacrine and horizontal cells were generated *in vivo* and GCs in cell culture. In addition, Ikaros mis-expression caused a reduction in late-born neurons (bipolar cells) and prevented Müller glia formation (**Figure 2A**). Consistent with this, Ikaros-deficient retinas exhibited a permanent reduction in most early-born cell types. Cones were not affected by the gain or loss of Ikaros, suggesting that different regulatory mechanisms control the timing of their production (Elliott et al., 2008). These findings indicate that Ikaros is required for progression to a late temporal state. Surprisingly, the timing of Ikaros activation is due to regulated translational repression, because Ikaros mRNA is expressed throughout retinal development, whereas the protein is present only in early RPCs (**Figure 2A**). Although not currently proven, key mediators of this repression might be miRNAs, as suggested by the similarity of the phenotypes observed after Ikaros mis-expression and Dicer CKO by αPax6-Cre transgene (see above).

A central role of Ikaros in determining the temporal fate of neurons in mouse was recently indicated also by a study of cortical development. Ikaros is expressed in progenitor cells of the mouse cerebral cortex at high level during the early stages of neurogenesis and thereafter its expression decreases over time. Sustained Ikaros expression prolonged the period of the generation of deep-layer neurons and delayed the production of late-born neurons. However, there is no direct evidence that Ikaros expression during corticogenesis is regulated at the post-transcriptional level as in the developing retina. In fact, Ikaros mRNA level is high at early stages and decreases by over 80% from embryonic E10.5 to E15.5. A possible role of miRNA in mediating the decrease of Ikaros mRNA level during cortical development was discussed (Alsiö and Tarchini, 2013).

Distinct miRNAs that can rescue Pax6-Cre driven Dicer CKO have recently been found. These miRNAs, let7, miR-9, and miR-125, are expressed in early retinal progenitors and serve as key regulators of the early to late developmental transition in retinal progenitors. When down-regulated, they cause an increase in GCs, whereas their up-regulation accelerates retinogenesis, increasing the ratio of late photoreceptor cells (rods) at the expense of early neurons (ganglion and horizontal cells). Let7, miR-9, and miR-125 target Protogenin (Prtg) and Lin-28b, two proteins that are crucial for maintaining an early competence state of RPCs. In fact, over-expression of Prtg and Lin-28b from E16 caused an extra number of heterochronic GCs that were generated at late times in retinogenesis (**Figure 2A**). Ikaros and Lin-28/Prtg seem to constitute two parallel pathways for the control of developmental timing, because let7, miR-9, and miR-125 do not appear to directly regulate the expression of Ikaros. However, there are conserved binding sites for miR-125 in the 3′UTR of two members of the Ikaros family, Ikzf3 and Ikzf5. These two genes show small increases of expression in the Dicer-CKO retina and the possibility that they play a role in retinal development has to be considered (La Torre et al., 2013).

Finally, key transcription factors of late retinal cell identity that are regulated at the translational level have been described in *Xenopus*. Xotx5b is the *Xenopus* homolog of the mammalian homeobox gene Crx and specifies photoreceptor identity. Xotx2 and Xvsx1 are the *Xenopus* counterparts of the mammalian Otx2 and Vsx2 homeobox genes, respectively, and support the differentiation of bipolar cells in *Xenopus* (Viczian et al., 2003; D’Autilia et al., 2006; Decembrini et al., 2006). Xotx5b, Xvsx1, and Xotx2 are transcribed since the early stages of retinogenesis in multipotent progenitor cells, but their translation is inhibited until later stages, when the generation of photoreceptor and bipolar cells begins. This translational inhibition is due to signals in the 3′UTR and is controlled by progression of the cell cycle (Decembrini et al., 2006). We have identified a set of four miRNAs that inhibit the translation of Xvsx1 and Xotx2 by binding to their 3′UTR. The four miRNAs (miR-129, miR-155, miR-214, and miR-222) are down-regulated as retinal development proceeds. Interestingly, their expression is decreased in early progenitors by the inhibition of the Shh pathway, which has the effect of lengthening the cell cycle, and is increased in progenitors forced into the S-phase. These treatments, respectively, accelerate and block the translation of Xvsx1 and Xotx2. We have proposed that cell cycle length, which is known to increase as retinogenesis progresses (Alexiades and Cepko, 1996), provides an intrinsic timer that regulates cell birth through miRNA activity (Decembrini et al., 2009; Pitto and Cremisi, 2010; **Figure 2B**). Shh is a possible mediator of this process, as it regulates the cell cycle length in the retina (Locker et al., 2006).

## CONCLUSION

The generation of distinct types of neurons in the cerebral cortex and neural retina relies on the ordered activation of cell fate genes over time. Studies in *Xenopus* and mouse retinal development described key proteins of neuronal identity whose expression is regulated at the translational level. Distinct miRNAs target these proteins and are crucial for early or late competence of progenitor cells in retinogenesis. Although no specific miRNA has been found to control the translation of key factors of cell fate in the cortex, the involvement of miRNAs in the control of the competence of cortical progenitor cells (CPCs) is strongly suggested by the results of Dicer down-regulation in CKO mice. In both the retina and cortex, expression of miRNAs is necessary for the transition from early to late development. However, in *Xenopus* retinogenesis there is evidence that distinct miRNAs must also be down-regulated to generate the latest neuron types.

An intriguing hypothesis is that the multipotency of early progenitor cells results from the transcription of mRNAs that serve to specify different neuronal identities, but are repressed by miRNAs. The release from the translational inhibition of distinct types of such mRNAs might determine what type of neuron is generated, and when. In *Xenopus*, release from the translational inhibition of Xvsx1 and Xotx2 is due to cell cycle lengthening, which causes the down-regulation of the four miRNAs targeting Xvsx1 and Xotx2. A similar mechanism, which makes use of cell-cycle-dependent miRNAs, might provide an intrinsic timer to regulate the cell birth of different types of neurons (**Figure 2B**). Shh, which regulates the cell cycle length in both the cortex and retina, might play a key role in this regard, and its function in temporally regulated aspects of retinogenesis and corticogenesis warrants further study.

## Conflict of Interest Statement

The author declares that the research was conducted in the absence of any commercial or financial relationships that could be construed as a potential conflict of interest.
